# Identification of a novel Rev-interacting cellular protein

**DOI:** 10.1186/1471-2121-6-20

**Published:** 2005-04-24

**Authors:** Susanne Kramer-Hämmerle, Francesca Ceccherini-Silberstein, Christian Bickel, Horst Wolff, Michelle Vincendeau, Thomas Werner, Volker Erfle, Ruth Brack-Werner

**Affiliations:** 1 Institute of Molecular Virology, GSF-National Research Center for Environment and Health, Ingolstädter Landstraße 1, D-85764 Neuherberg, Germany; 2 Department of Experimental Medicine, University of Rome Tor Vergata, Via Montpellier 1, Rome 00133, Italy; 3 Genomatix Software GmbH, Landsbergerstr. 6, D-80339 München, Germany

## Abstract

**Background:**

Human cell types respond differently to infection by human immunodeficiency virus (HIV). Defining specific interactions between host cells and viral proteins is essential in understanding how viruses exploit cellular functions and the innate strategies underlying cellular control of HIV replication. The HIV Rev protein is a post-transcriptional inducer of HIV gene expression and an important target for interaction with cellular proteins. Identification of Rev-modulating cellular factors may eventually contribute to the design of novel antiviral therapies.

**Results:**

Yeast-two hybrid screening of a T-cell cDNA library with Rev as bait led to isolation of a novel human cDNA product (16.4.1). 16.4.1-containing fusion proteins showed predominant cytoplasmic localization, which was dependent on CRM1-mediated export from the nucleus. Nuclear export activity of 16.4.1 was mapped to a 60 amino acid region and a novel transport signal identified. Interaction of 16.4.1 with Rev in human cells was shown in a mammalian two-hybrid assay and by colocalization of Rev and 16.4.1 in nucleoli, indicating that Rev can recruit 16.4.1 to the nucleus/nucleoli. Rev-dependent reporter expression was inhibited by overexpressing 16.4.1 and stimulated by siRNAs targeted to 16.4.1 sequences, demonstrating that 16.4.1 expression influences the transactivation function of Rev.

**Conclusion:**

These results suggest that 16.4.1 may act as a modulator of Rev activity. The experimental strategies outlined in this study are applicable to the identification and biological characterization of further novel Rev-interacting cellular factors.

## Background

The human immunodeficiency virus (HIV) Rev protein is a small (116 amino acids) post-transcriptional activator of expression of incompletely spliced and unspliced HIV mRNAs. Since these HIV transcripts direct production of progeny virions, Rev is a crucial factor in HIV replication (for overview see [1]). Rev interacts with HIV mRNAs by binding to a structured RNA element called the RRE (Rev response element). Rev offsets the activities of inhibitory sequences (INS) in HIV-1 mRNAs [2,3] and promotes their export to the cytoplasm. Once in the cytoplasm, Rev may also stimulate production of viral proteins on translational level (reviewed in [4]).

Rev characteristically localizes to the nucleus, where it accumulates in nucleoli. However, a proportion of the Rev molecules expressed in a cell continuously shuttles between nucleus and cytoplasm by using active transport mechanisms both for entry into and exit from the nucleus.

**Figure 1:**
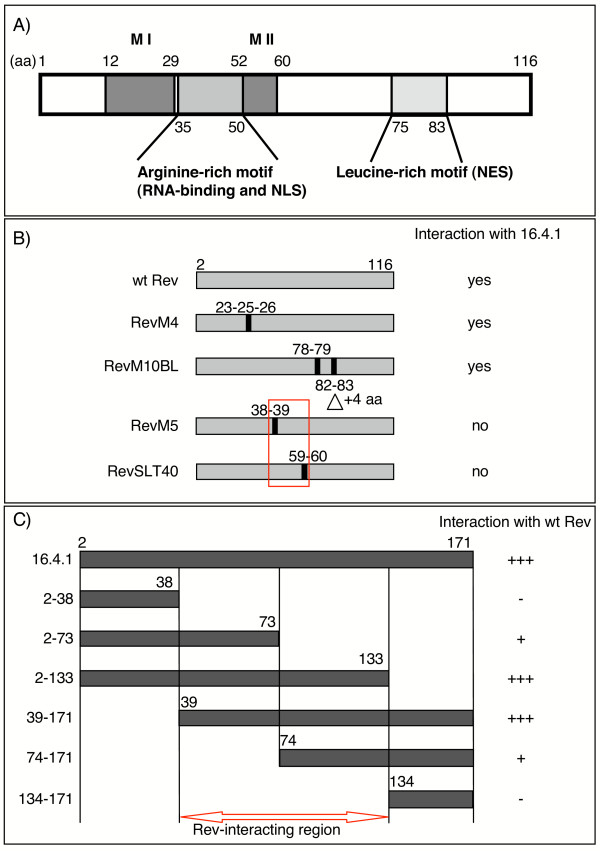
**Analysis of interaction of 16.4.1 with Rev**. (A) Schematic overview of domains of Rev. Locations of functional regions are taken from [5]. Numbering of amino acids (aa) is based on the Rev sequence of HIV-1 isolate HXB-2. MI and MII: Regions that direct multimerization of Rev. NLS: nuclear localization signal; NES: nuclear export signal. (B) Identification of amino acid positions in Rev required for interaction with 16.4.1. Bait proteins containing Rev or various mutants of Rev were analysed for interaction with 16.4.1-prey. Numbers indicate positions of amino acid changes in Rev mutants. Mutations are as follows: RevM4: Y^23 ^to D^23^, S^25 ^to D^25^, N^26 ^to L^26 ^[45]; Rev M10BL: L^78 ^to D^78^, E^79 ^to L^79 ^and insertion of EDLP between L^81 ^and T^82 ^[47]; Rev M5: R^38 ^to D^38^, R^39 ^to L^39 ^[45]; RevSLT40: I^59 ^to D^59^, L^60 ^to D^59 ^[46]. Interaction is indicated by growth of yeast transformants under selective conditions (≥ 500 transformants per plate). Results represent four independent experiments. The red box marks the amino acid positions in Rev required for interaction with 16.4.1 and the location of the putative 16.4.1-interaction region of Rev (aa 38 to 60). (C) Identification of regions of 16.4.1 required for interaction with Rev. Prey proteins containing various regions of the 16.4.1 domain were analysed for interaction with Rev-bait. Interaction was analysed by growth of yeast transformants under selective conditions. Results represent three independent experiments. +++ 700–1400 transformants per plate; + 200–400 transformants per plate; – no transformants. 16.4.1 regions comprising amino acids 2 to 133 and 39 to 171 interacted with Rev with similar efficiency as full-length 16.4.1, whereas regions 2 to 73 and 74 to 171 showed weaker interaction. No interaction was observed with regions 2 to 38 and 134 to 171. The red arrow indicates the putative Rev-interaction region of 16.4.1 (aa 39 to 133).

Mutational analyses of the Rev protein have identified various functionally important regions, indicating that Rev is organized into modular domains (Fig. 1A; reviewed in [5] and [6]). The N-terminal domain of Rev contains an arginine-rich motif (ARM; amino acids 35 to 50) with dual functions as a nuclear localization signal (NLS) and RNA binding domain. Sequences flanking the ARM (amino acid regions 12 to 29 and 52 to 60) direct multimerization of Rev. The C-terminal domain of Rev, also known as activation domain, contains a leucine-rich motif (amino acid region 75 to 83) that functions as a nuclear export signal.

Biochemical analyses indicate that Rev directly binds the nuclear transport receptors Importin β and CRM1/Exportin 1 [7-9]. Interaction of Rev with CRM1/Exportin 1 was confirmed by two-hybrid assays in yeast [10] and in human cells [11,12]. Together with results from various other experimental approaches (reviewed in [6]), these observations have led to the concept that import of Rev into the nucleus is mediated by interaction of the ARM/NLS with Importin  and export of the Rev-RNA complex from the nucleus by interaction of the Rev-NES with CRM1/Exportin 1.

Various other Rev-interacting cellular factors have been identified by using Rev or segments of Rev for yeast two-hybrid screening of cDNA libraries or for biochemical purification of interacting factors from cell extracts.

Cellular factors shown to interact with the ARM of Rev include p32 [13,14] and B23 [15,16]. Human p32 was recently reported to block splicing of Rev-dependent HIV transcripts [17]. The nucleolar protein B23 was shown to stimulate nuclear import of Rev [18] and counteract aggregation of Rev [19]*in vitro*.

The C-terminal domain of Rev interacts with various human nucleoporins, including hRIP/hRab, NLP-1, Nup98, and Nup214 [20-26]. Other factors shown to interact with this domain of Rev are eIF-5A [27] and the nuclear kinesin-like protein REBP [28]. hRIP/hRab, Nup 98 and eIF-5A interact with CRM1 as well as Rev [10,25,26,29], suggesting that Rev can associate with CRM1 in multifactorial complexes in which CRM1 "bridges" the interaction of Rev with other factors. Rev-CRM1 complexes containing hRIP/hRab or eIF-5A may be crucial for Rev-dependent export of HIV RNAs, since eIF-5A and hRIP/hRab have been shown to be essential for Rev-directed RNA export in *Xenopus *oocytes and in human cells, respectively [30,31].

Nuclear export of Rev has proven to be exemplary for many viral and cellular factors (reviewed in [32,33]). Since the discovery of leucine-rich signals in Rev [34] and in the cellular regulatory factor PKIα [35], these sequences have been shown to mediate the export of numerous factors from the nucleus by CRM1/Exportin1 [36]. The drug Leptomycin B (LMB), first shown to block nuclear export of Rev [37], proved to be a potent inhibitor of CRM1-dependent export [38,39] and is now widely used to identify transport substrates of CRM1. Elucidating interactions of Rev with cellular factors is highly relevant to understanding pathogenicity of HIV and may have an impact on the design of therapeutic anti-HIV strategies. The functional diversity of Rev and its activities in both nuclear and cytoplasmic compartments of the cell suggest the existence of still unidentified Rev-interacting factors. Therefore we reasoned that screening of a human cDNA library with Rev as "bait" should lead to isolation of novel Rev-interacting human factors. Of particular interest would be the identification of unknown human gene products, since their interaction Rev would not only be relevant for Rev function but would also provide a key for biological characterisation of these novel factors.

Here we identify a human cDNA that encodes a novel protein (16.4.1) that interacts specifically with Rev via sequences in the N-terminal half of Rev. We show that 16.4.1 is exported from the nucleus by CRM1 and localizes to the cytoplasm. In Rev-expressing cells, 16.4.1 is recruited to nucleoli. 16.4.1 has a negative effect on Rev function in a Rev-reporter assay. These results suggest that 16.4.1 can act as a modulator of Rev function.

## Results

### Identification of novel HIV-1 Rev-interacting proteins

To identify novel Rev-interacting proteins, we screened a library of cDNAs derived from the human Jurkat T-cell line with full-length Rev as bait in a yeast two-hybrid system. Repeated selection procedures led to isolation of two library plasmids (11.5.1 and 16.4.1) encoding specific interactors of Rev.

Sequence analyses and data base comparisons revealed that the 936 bp insert in plasmid 11.5.1 is identical with a segment of a 1543 bp cDNA encoding human DNA binding protein B (dbpB; NCBI accession number BC002411) [40]. The predicted coding sequences in the 11.5.1 cDNA comprise the C-terminal 139 amino acids of the dbpB protein (324 amino acids; NCBI accession number M24070). Several biological activities have been attributed to dbpB, including binding to DNA [41] and RNA [42,43] and regulation of transcription [44].

The other library plasmid 16.4.1 contained a 696 bp insert of which a region of over 450 nucleotides showed strong similarity to a sequence within a human fetal heart cDNA (NCBI accession number W67699). In the fetal heart cDNA the matching region encompasses a predicted open reading frame. Alignment of the 16.4.1 and the fetal heart cDNA sequences yielded a sequence encoding a hypothetical 171 amino acid 16.4.1 protein. Since interaction with Rev is the first biological activity associated with this gene product, we analysed interaction of Rev with the 16.4.1 protein in more detail.

To investigate which regions of Rev contribute to interaction with the 16.4.1 protein, we analysed the capacity of various known mutants of Rev to interact with 16.4.1 in the yeast two-hybrid assay. The amino acid exchanges in these mutants map to regions associated with major biological properties of Rev (Fig. 1B), including multimerization (RevM4 [45] and RevSLT40 [46]), RNA binding and nuclear localization/accumulation (RevM5 [45]) and nuclear export of Rev (RevM10BL [47]). Expression of LexA-Rev-mutant bait proteins in yeast transformants was confirmed by Western blot analysis with polyclonal antibodies against Rev (data not shown). As positive control for Rev interaction, interaction analysis was performed with LexA-Rev bait and B42AD-Rev prey, confirming oligomerization of wildtype Rev molecules with each other (data not shown).

While Rev mutants RevM4 and Rev M10BL were capable of interacting with 16.4.1, no interaction was observed with Rev mutants RevM5 and RevSLT40 (Fig. 1B). These results indicate that amino acid residues R^38 ^or R^39 ^of the ARM and I^59 ^or L^60 ^of the multimerization region II (MII) are required for interaction of Rev with the 16.4.1 protein. Furthermore, they suggest that the 16.4.1 interacting sequences in Rev are located between aa positions 38 and 60.

For more detailed study of the interaction of the 16.4.1 protein with Rev, yeast two-hybrid analysis was performed with various segments of the 16.4.1 cDNA as prey and wildtype Rev as bait (Fig. 1C). Amino acid regions of 16.4.1 extending from position 2 to 133 and from position 39 to 171 showed similar Rev-binding capacity as full-length 16.4.1 protein. In contrast, both the N-terminal region (2 to 38) and the C-terminal region (134 to 171) of 16.4.1 failed to interact with Rev. While 16.4.1 protein fragments from position 2 to 73 or position 74 to 171 clearly interacted with Rev, interactions were weaker than that of full-length 16.4.1. These results indicate that the Rev-interacting region of the 16.4.1 protein is located between amino acid positions 39 and 133 and that, within this region, sequences N- and C-terminal of position 73 contribute to interaction with Rev.

### Interaction of the 16.4.1 protein with Rev, CRM1 and itself in human cells

The interaction of the 16.4.1 protein with Rev in yeast raises the question whether the 16.4.1 protein can also interact with Rev in human cells. It was also of interest whether 16.4.1 is capable of interacting with human CRM1, since CRM1 has been shown to interact with several Rev-associated factors (see Background).

**Figure 2:**
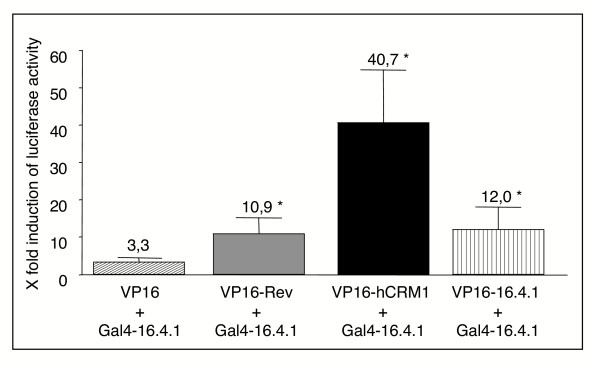
**Interaction of 16.4.1 with HIV-1 Rev, hCrm1 and with itself in human cells**. 16.4.1 interactions in human cells were analysed with a mammalian two-hybrid assay in which the interaction of a protein fused to the Gal4 DNA-binding domain with a second protein fused to the VP16-activator domain induces transcription of a luciferase reporter gene. HEK293 cells were cotransfected with pBIND-16.4.1 plasmid for expression of Gal4-16.4.1 fusion protein, a pACT plasmid for expression of the indicated VP16-fusion protein and with the pG5*luc *reporter plasmid. Parallel cultures were cotransfected with pG5*luc *and the pBIND and pACT vectors to determine basal expression of the luciferase gene. Cells were lysed 48 hours after transfection and luciferase activities determined. Bars indicate the mean fold- induction of luciferase activity over basal expression ± SEM (standard error of the mean) and represent at least 6 independent transfection experiments. Cells coexpressing Gal4-16.4.1 and VP16 fused with Rev (grey bar), hCRM1 (black bar) or 16.4.1 (vertically striped bar) domains showed significantly stronger induction of luciferase production than cells coexpressing Gal4-16.41 and unfused VP16 (diagonally striped bar). Statistical analysis was performed by two-tailed Mann-Whitney U test.

We addressed these issues with a mammalian two-hybrid assay, in which the interaction of a protein fused to the Gal4 DNA-binding domain with a second protein fused to the VP16-activator domain induces transcription of a luciferase reporter gene from a synthetic promoter (for details see Materials and Methods). Rev was fused to VP16 (VP16-Rev) to avoid unspecific interactions between the acidic VP16 domain [48] and the basic Rev protein (estimated pI = 9.93; MacVector calculation). Functionality of VP16-Rev was demonstrated (data not shown) in a Rev-reporter assay [3]. For interaction analysis, HEK293 cells were cotransfected with expression plasmids for VP16-Rev and Gal4-16.4.1 fusion proteins and the reporter plasmid pG5*luc*. As shown in Fig. 2, a ≈11-fold mean induction of luciferase activity was observed in 14 independent transfection experiments. Assessment of interaction of 16.4.1 with human CRM1 in cells coexpressing Gal4-16.4.1 and VP16-hCRM1 revealed a ≈41-fold mean induction of luciferase activity export (n = 7) (Fig. 2). Self-interaction of the 16.4.1 domain was analysed by coexpressing Gal4-16.4.1 and VP16-16.4.1, resulting in ≈12-fold mean induction of luciferase activity (n = 6).

In all three cases, induction of luciferase activity was significantly (p < 0.04) increased over induction levels obtained in control assays with unfused VP16 and Gal4-16.4.1 (3.3-fold; n = 7).

These results indicate that the 16.4.1 domain is capable of interacting with Rev as well as with the export receptor CRM1 and of forming homo-oligomers in human cells.

### Cytoplasmic localization of 16.4.1 is CRM1/Exportin 1 dependent

**Figure 3:**
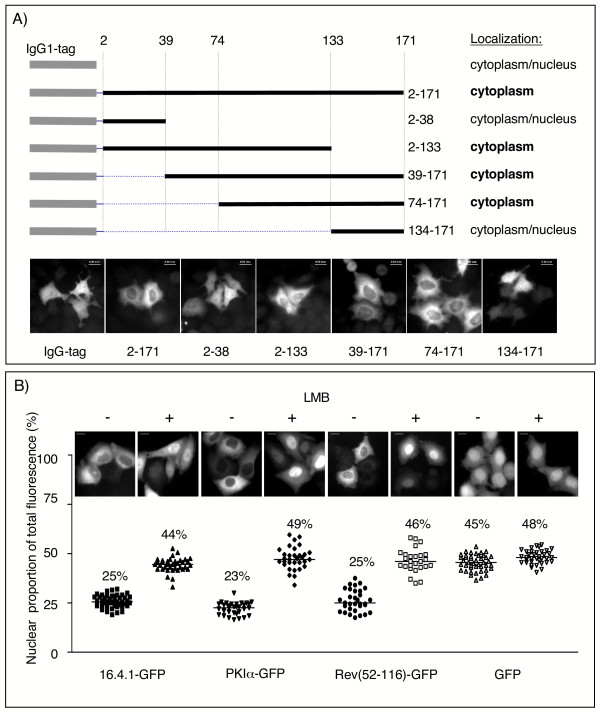
**CRM1-dependent cytoplasmic localization of 16.4.1**. HeLa cells were transfected with plasmids directing expression of IgG1-16.4.1 or 16.4.1-GFP fusion proteins and subcellular distribution of tagged proteins analysed 24 hours later in fixed cells. (A) Subcellular distribution of IgG1 fusion proteins containing full length 16.4.1 or various segments of 16.4.1. IgG1-16.4.1 proteins were detected by immunocytochemistry with a Cy3-conjugated anti human IgG1 antibody. A schematic diagram of the IgG1-16.4.1 fusion proteins and a summary of their localization behavior are shown at the top. Representative images of cells expressing IgG1 fusion proteins containing the indicated amino acid regions of 16.4.1 are shown below. IgG1 fusion proteins containing amino acids 2–171 (full-length), 2–133, 39–171 and 74–171 of 16.4.1 were predominantly cytoplasmic, whereas fusion proteins with amino acids 2–38 or 134–171 and unfused IgG1 were both cytoplasmic and nuclear. Scale bars: 20 μm. (B) Disruption of predominantly cytoplasmic localization of 16.4.1-GFP by treatment of cells with the CRM1-inhibitor Leptomycin B (LMB). HeLa cells were transiently transfected with plasmids for expression of 16.4.1-GFP, PKIα-GFP, Rev(52–116)-GFP and unfused GFP. Cells were treated with LMB (5 nM) for two hours. Representative images of the subcellular distribution of the GFP fusion proteins in untreated (-) and LMB treated cells (+) are shown at the top. Symbols in the graph indicate the nuclear proportion of fluorescence (%) in individual cells and horizontal lines and numbers the median of the cell population. LMB treatment increased the median nuclear proportion of 16.4.1-GFP from 25% to 44%. LMB had a similar effect on localization of GFP fusion proteins containing PKIα or the carboxyterminal region of Rev (aa 52–116), which are known transport substrates of CRM1. In contrast, LMB only marginally affects subcellular distribution of unfused GFP. Scale bars: 10 μm.

Comparison of the sequence in the 16.4.1 cDNA with the fetal heart cDNA indicated that the 16.4.1 sequence was incomplete at its 5' terminus. To generate a full-length (171 aa) 16.4.1 coding sequence, nucleotides encoding the first 8 N-terminal amino acids derived from the predicted open reading frame of the fetal heart cDNA were inserted upstream of the 16.4.1 cDNA. To analyse subcellular localization of the 16.4.1 protein, cells were transfected with plasmids directing expression of fusion proteins containing full-length 16.4.1 or various segments of 16.4.1. Those fusion proteins contained either a N-terminal IgG1 tag or a C-terminal GFP tag. The full-length IgG1-16.4.1 fusion protein (IgG1-2-171) was located mainly in the cytoplasm of HeLa cells (Fig. 3A). IgG1 fusion proteins with 16.4.1 regions extending from amino acid position 2 to 133, 39 to 171 and 74 to 171 showed similar predominantly cytoplasmic localization (Fig. 3A). In contrast, IgG1 fusion proteins with the N-terminal region (2 to 38) or the C-terminal region (134 to 171) of 16.4.1 were apparent in both nucleus and cytoplasm, similar to unfused IgG1. These results demonstrate that the 16.4.1 protein is capable of cytoplasmic accumulation and suggest that sequences directing cytoplasmic localization of the 16.4.1 protein are located between amino acid positions 74 to 133.

The 16.4.1-GFP fusion protein showed similar cytoplasmic localization as IgG1-16.4.1 (Fig. 3B). Quantitative evaluation of subcellular distribution of GFP fluorescence [49] revealed that only 25% of total fluorescence was contained in the nuclei of 16.4.1-GFP expressing cells. This localization is comparable to that of GFP fusion proteins containing PKIα (PKIα-GFP) or the carboxyterminal half of Rev (Rev(52–116)-GFP), which localize to 23% and 25%, respectively, in the nucleus (Fig. 3B). PKIα and the carboxyterminal half of Rev contain well-characterized recognition signals for CRM1/Exportin 1-dependent export [36]. Similar cytoplasmic localization of 16.4.1-GFP and interaction of 16.4.1 with CRM1/Exportin 1 in human cells (Fig. 2) raised the possibility that cytoplasmic localization of 16.4.1-GFP at steady state may involve nuclear export of 16.4.1 by CRM1/Exportin 1. Therefore we analysed the effect of Leptomycin B (LMB), an inhibitor of CRM1-dependent nuclear export [11,37,38] on subcellular distribution of 16.4.1-GFP. LMB treatment significantly increased the nuclear proportion of 16.4.1-GFP from 25% to 44%. LMB-induced nuclear redistribution was similar in cells expressing PKIα-GFP and Rev(52–116)-GFP, whose nuclear proportion increased to 49% and 46%, respectively. Quantitative analysis demonstrated that 45% of unfused GFP localized to the nucleus, in agreement with its known capacity to diffuse throughout the cell [50]. LMB had no significant effect on subcellular distribution of unfused GFP.

These results indicate that cytoplasmic localization of 16.4.1 involves nuclear export by CRM1/Exportin1. Amino acid region 74 to 133 of 16.4.1 seems to be crucial for those transport processes.

### Identification of a candidate nuclear export signal in 16.4.1

**Figure 4:**
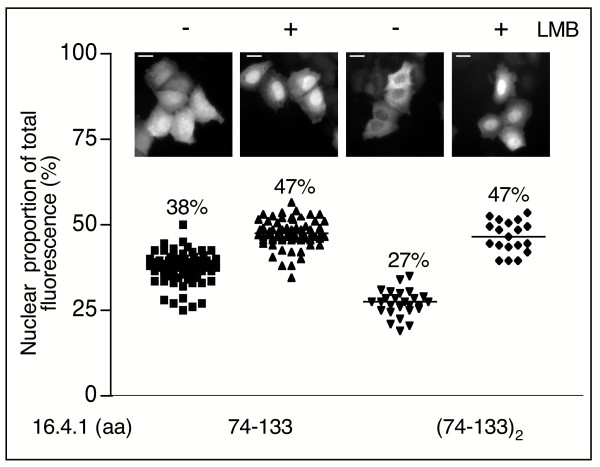
**Analysis of CRM1-dependent nuclear export of amino acid region 74 to 133 of 16.4.1**. HeLa cells were transfected with plasmids directing expression of GFP fusion proteins containing 16.4.1 region 74–133 in single copy or in tandem (74–133)_2_. Representative images of the subcellular distribution of the GFP fusion proteins in untreated (-) and LMB treated cells (+) are shown at the top. Symbols in the graph indicate the nuclear proportion of fluorescence (%) in individual cells and horizontal lines and numbers the median of the cell population. Scale bars: 10 μm. Inhibition of CRM1 by LMB treatment increases nuclear proportion of GFP fusion proteins containing aa residues 74 to 133 of 16.4.1. GFP fusion proteins containing two copies of region 74 to 133 show stronger cytoplasmic localization than GFP fusion proteins with a single copy. These results indicate that region 74 to 133 of 16.4.1 is a substrate for CRM1-dependent export, which is recognized more efficiently in tandem than as a single copy.

To further characterize the involvement of the amino acid region 74–133 in cytoplasmic localization of 16.4.1, we assessed subcellular distribution of GFP fusion proteins containing this region of 16.4.1. Cells expressing a GFP fusion protein with a single copy of aa 74–133 of 16.4.1 contained a higher proportion of nuclear fluorescence (38%, Fig. 4) than cells expressing GFP fusion proteins with full-length 16.4.1 (25%, Fig. 3B). However, GFP-fusion proteins containing two copies of region 74 to 133 of 16.4.1 in tandem showed similar cytoplasmic localization (Fig. 4; 27% nuclear proportion) as full-length 16.4.1-GFP (Fig. 3B; 25%). Treatment of cells with LMB raised nuclear proportions of GFP fusion proteins with one or two copies of 16.4.1 region 74–133 to similar levels as full-length 16.4.1-GFP. These results suggest that the region between amino acid positions 74 and 133 contains a CRM1/Exportin 1 dependent nuclear export signal, which can act in a cumulative manner.

**Figure 5:**
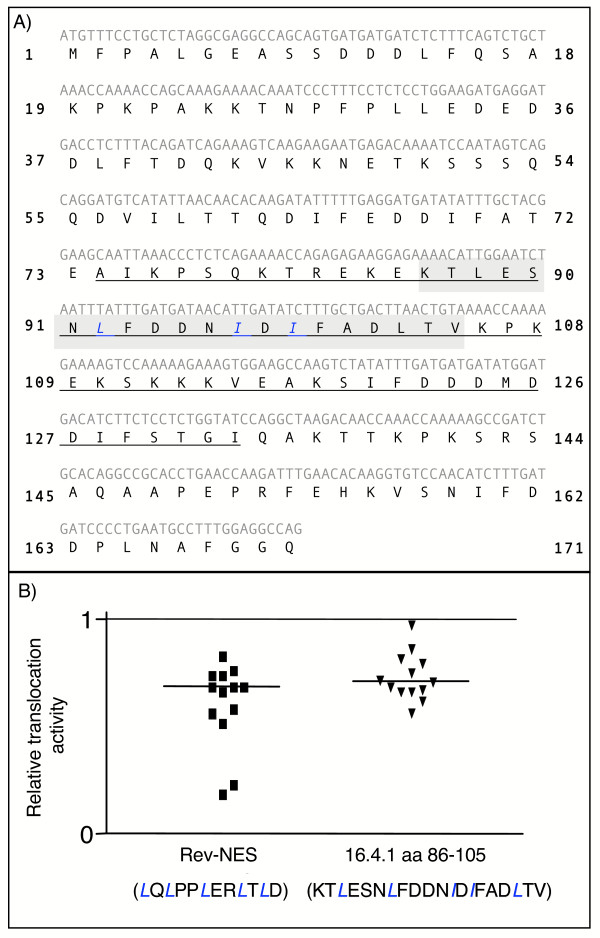
**Functional analysis of a nuclear export signal in 16.4.1**. (A) Depicted is the sequence (nucleotides and predicted amino acids) of the 16.4.1 protein investigated here. The sequence encoding amino acids 1–8 are derived from the fetal heart cDNA W67699. The region between amino acid residue 74 and 133 showing CRM1-dependent nuclear export activity (Fig. 4) is underscored. The amino acid sequence between residues 86 and 105 (shaded in grey) contains several Leucine and Isoleucine residues representing a candidate nuclear export signal. (B) Functional characterisation of the Leucine-Isoleucine rich sequence of 16.4.1. Comparison of the translocation activities of 16.4.1 region 86–105 and the Rev-NES in a microinjection-based transport assay [51]. Transport substrates were generated by conjugating peptides containing region 86–105 of 16.4.1 or region 73–84 of Rev with bovine serum albumin (BSA) labeled with a red fluorescent dye. Transport substrates were coinjected into the nucleus with an injection control consisting of unconjugated BSA labeled with a different fluorescent dye (e.g. green). The proportion of each fluorescent label in the nucleus of the injected cell was determined and the ratio of fluorescence of the transport substrate to fluorescence of the injection control calculated. This ratio represents the relative translocation activity of the transport substrate and is indicated in the graph. Nuclear export activity yields ratios <1 as demonstrated for the Rev NES. Transport substrates containing amino acid region 86–105 of 16.4.1 also yielded a relative translocation activity <1, indicating that this region of 16.4.1 can function as a nuclear export signal.

Examination of the hypothetical amino acid sequence of region 74 to 133 revealed a clustering of leucine and isoleucine residues between amino acid 86 and 105 (Fig. 5A, shaded in grey). To analyse whether region 86 to 105 of the 16.4.1 protein functions as a nuclear export signal, we compared its translocation capacities with the Rev-NES in a previously described microinjection assay [51] (Fig. 5B). In this assay, peptides bearing the candidate transport sequences are linked to fluorescently labeled bovine serum albumin (BSA). These potential transport substrates are coinjected into the nucleus with unlinked BSA labeled with a different fluorescent color that serves as injection control. Two hours later, cells are fixed and the percentage of each fluorescent label in the nuclear compartment of individual cells determined. The relative translocation activity signifies the ratio of fluorescence of the transport substrate to the fluorescence of the injection control. Selective export of the transport substrate from the nucleus yields relative translocation activities < 1, as demonstrated for a transport substrate containing the NES of Rev (Fig. 5B and [51]). A substrate containing the 16.4.1-derived sequence also yielded a relative translocation activity < 1 (Fig. 5B). These results indicate that region 86 to 105 of 16.4.1 sequence can function as a nuclear export signal.

**Figure 6:**
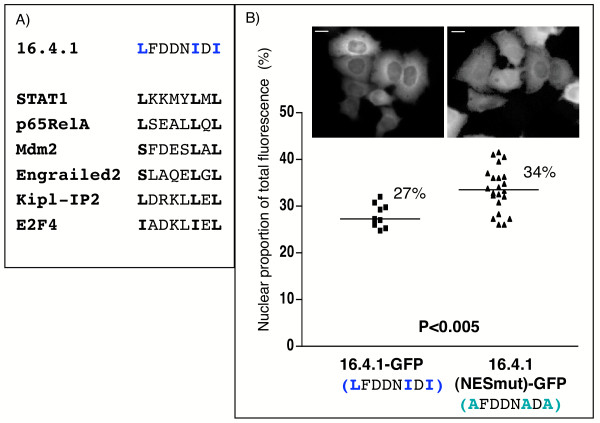
**Influence of the nuclear export signal on cytoplasmic localization of 16.4.1**. (A) Sequences identified in 16.4.1 and in proteins contained in a database of experimentally verified NES by a common weight matrix. Protein sequences in NESbase version 1 [36] were analysed with a collection of 7 weight matrices. These identified 48 of 75 NES in their native protein context at default threshold (0.84). Analysis of the 16.4.1 sequence with reduced threshold settings (0.74) yielded a single match with matrix M5 but not with any other matrix. This match identified the 16.4.1 core NES (shown at top). Sequences in proteins of the NES database identified by M5 at default threshold are shown below. All sequence matches map to experimentally verified NES. (B) To assess functionality of the novel 16.4.1 NES in the context of the full length protein, a 16.4.1 mutant was generated in which L^92^, I^97 ^and I^99 ^were replaced by alanine residues. HeLa cells were transfected with plasmids for expression of GFP fusion proteins containing wild type (16.4.1-GFP) or mutated (16.4.1(NESmut)-GFP) 16.4.1 and subcellular distribution of GFP fusion proteins were evaluated by quantitative fluorescence microscopy. Cells expressing 16.4.1(NESmut)-GFP showed a significantly higher nuclear proportion of fluorescence (34%) than cells expressing wild type 16.4.1-GFP (27%). This effect was significant (p < 0.005). Scale bars: 10 μm.

To further characterize this nuclear export signal in 16.4.1 we took advantage of a collection of weight matrices (M1-M7) derived for recognition of NES by bioinformatics (Blossom similarity matrix). These matrices recognized 48 out of 75 signals of a published NES database [36] at a default threshold of 0.84 in the context of their native proteins. No match was obtained upon scanning of the 16.4.1 amino acid sequence with these matrices at default threshold. This indicates that the 16.4.1 sequence is distinct from the 48 NES represented by the matrices. However, rescanning of the 16.4.1 sequence at a lower threshold (0.74) yielded a single match for matrix M5 (0.78), comprising amino acids 92–99 of 16.4.1 (core NES). At default threshold the same matrix recognized a specific group of NES that includes the NES of Stat1 and p65RelA (Fig. 6A). However this matrix did not recognize the NES of PKIα or Rev, which were recognized by different matrices. An artificial 16.4.1 NES sequence containing leucine instead of isoleucine residues at positions 99 and 101 was recognized by matrix M5 above default score (0.86) but by no other matrices, even at reduced thresholds.

Finally we investigated whether the candidate transport signal also shows nuclear export activity in the context of the complete 16.4.1 protein. As shown in figure 6B, the leucine and two isoleucine residues of the 16.4.1 core NES were changed to Alanin and the subcellular distribution of the 16.4.1(NESmut)-GFP was compared to the wildtype 16.4.1 fused to GFP. The mutant 16.4.1-GFP fusion protein localized to significantly higher levels in the nucleus than wildtype 16.4.1-GFP (34% versus 27%). However, the nuclear proportion of the mutant 16.4.1-GFP remained below that of unfused GFP (Fig. 3B), indicating residual nuclear export of the mutant 16.4.1-GFP.

In summary, combined computational and functional analyses indicate that amino acid residues 86 to 105 act as a nuclear export signal, with amino acids 92 to 99 constituting a potential core NES. Mutational analysis indicates that the leucine/isoleucine of the 16.4.1 core NES contribute to but are not sole determinants of cytoplasmic localization of 16.4.1.

### Colocalization of 16.4.1 and Rev

This report demonstrates interaction of 16.4.1 and Rev in yeast and mammalian two-hybrid assays (Figs. 1 and 2). In these approaches, candidate interaction partners are artificially targeted to the nucleus to measure interaction-dependent reporter gene expression.

To analyse whether 16.4.1 and Rev interact under conditions in which they retain their natural localization behavior, we analysed cells coexpressing 16.4.1 and Rev for colocalization of both proteins.

To this end, we first established a HeLa cell line stably expressing 16.4.1-GFP and a corresponding control cell line expressing unfused GFP. The expression of 16.4.1-GFP for more than 20 passages did not affect cell growth monitored by measurement of growth curves and did not lead to cell toxicity detectable as release of lactate dehydrogenase (LDH) or ATP into cell culture supernatants (data not shown). Furthermore, long-term expression did not alter the predominantly cytoplasmic localization of 16.4.1-GFP.

**Figure 7:**
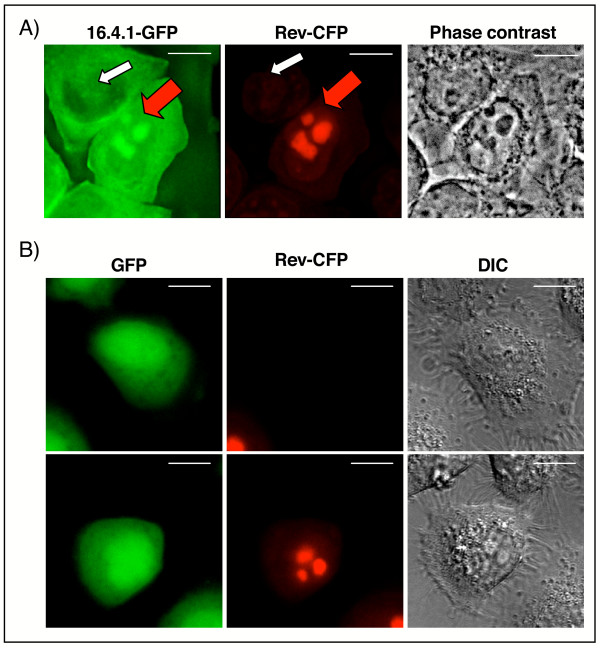
**Nucleolar colocalization of 16.4.1-GFP and Rev-CFP**. HeLa 16.4.1-GFP cells and control HeLa GFP cells were transiently transfected with a Rev-CFP expression plasmid. Nuclei were counterstained with Hoechst 33342. 3D-Maximum image projections of Z-stacks were deconvolved and processed by multichannel unmixing, applying identical parameters to all images (see Material and Methods). Left images represent the GFP-channel (green), middle images the CFP-channel (pseudocolored red) and right images either an interference contrast photo (A) or a single z-slice of a phase contrast image (B). (A) Exemplary images of over 25 analyzed cells demonstrating nucleolar colocalization of 16.4.1-GFP and Rev-CFP. The left image shows HeLa-16.4.1-GFP cells either without Rev-CFP (white arrow), or with concurrent Rev-CFP expression (red arrow). The nucleolar 16.4.1-GFP signal is only visible in cells coexpressing Rev-CFP. Cells with extremely high expression levels of Rev-CFP were excluded from analysis. Scale bars: 10 μm. (B) Controls for separation of GFP and CFP and signals by multichannel unmixing. Images of HeLa cells stably expressing GFP (HeLa-GFP) either alone or together with Rev-CFP are shown in the upper and lower panels, respectively. The intracellular distribution of the GFP signal is not influenced by coexpression of Rev-CFP. Conversely, a nuclear/nucleolar CFP signal is detected only in HeLa-GFP cells coexpressing Rev-CFP. These results confirm that multichannel unmixing eliminates spectral crosstalk between CFP and GFP channels. Scale bars: 10 μm.

For colocalization studies, HeLa 16.4.1-GFP cells and control HeLa-GFP cells were transfected with a plasmid directing expression of Rev-CFP (cyan fluorescent protein) fusion proteins. Transfected cells were subjected to epifluorescence microscopy and Z-stacks were collected. Images were processed by deconvolution and multichannel unmixing, allowing separate evaluation of the spatial distribution of GFP and CFP signals. Over 25 cells were analyzed. Multichannel unmixing is a recently developed technique for separate detection of fluorochromes that exhibit significant spectral overlap in conventional fluorescence microscopy setups, such as CFP and GFP (for a review see [52]). Fig. 7A shows examples of cells expressing 16.4.1-GFP either alone (white arrow) or together with Rev-CFP (red arrow). 16.4.1-GFP was only visible in the nucleoli of cells co-expressing Rev-CFP but not in cells lacking Rev-CFP (see also Fig. 3). Cells coexpressing 16.4.1-GFP and Rev-CFP showed stronger nucleoplasmic GFP fluorescence than HeLa 16.4.1-GFP cells lacking Rev-CFP. Rev-CFP retained typical nuclear/nucleolar localization [49] when coexpressed with 16.4.1-GFP, indicating that 16.4.1-GFP does not influence localization of Rev-CFP. Control imaging of HeLa cells expressing GFP either alone (Fig. 7B, top panel) or together with Rev-CFP (Fig. 7B, bottom panel) showed that presence of Rev-CFP did not influence the GFP signal and that the CFP signal was apparent only in cells expressing Rev-CFP. These results verified separation of Rev-CFP and GFP signals by the multichannel unmixing routine and confirmed that the CFP-tag in Rev-CFP does not affect localization of GFP.

These results indicate that Rev is capable of directing 16.4.1 to nucleoli and provide further evidence for interaction of Rev and 16.4.1 in human cells.

### Influence of 16.4.1 on Rev functions

**Figure 8:**
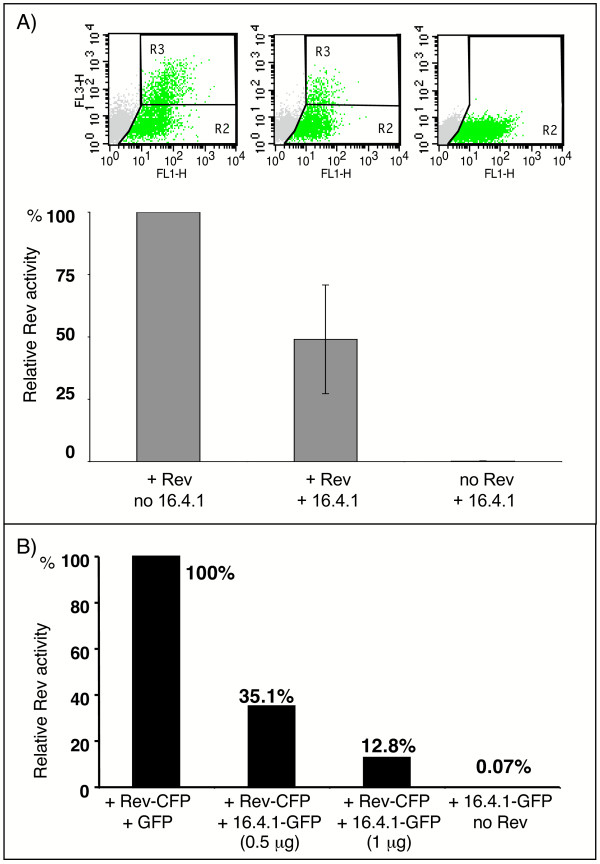
**16.4.1 influences transactivation capacity of Rev in human cells**. Panel (A) depicts the reduction of Rev activity obtained by cotransfection of 0.5 μg plasmids directing expression of 16.4.1 fusion proteins and 0.1 μg *rev *expression plasmids. The graph shows mean values and standard deviations of 5 independent experiments carried out in 293T cells expressing either IgG1-16.4.1 or 16.4.1-GFP in combination with Rev-GFP or Rev-CFP. FACS analysis was used to quantify the number of cells expressing the Rev-dependent RFP reporter encoded by pLRed(INS)_2_R (see Material and Methods) within the population of transfected cells. The dot plots above the bars (FL1 = green fluorescence, FL3 = red fluorescence) represent examples for evaluation of FACS data for fully functional Rev (left), inhibited Rev function by 16.4.1 coexpression (middle) and background expression of the reporter in the absence of Rev (right). The non-transfected, fluorescence negative 293T cell population is represented in grey and excluded from analyses. 293T cells containing the inactive reporter gene (no red fluorescence) and expressing GFP as transfection control were used to define the transfected cell population (R2, right dot plot). Cells transfected with all plasmids required for activation of reporter gene expression (expression plasmids encoding Rev, Tat and the reporter RFP) as well as for expression of the GFP transfection control contain a population of cells expressing both green and red fluorescent proteins defined as R3. The ratio of R3 to R2 represents the proportion of transfected cells showing Rev-dependent reporter expression (i.e. Rev activity). Rev activity in the absence of 16.4.1 was set at 100% (left bar of the graph). Coexpression of 16.4.1 fusion proteins diminished Rev activity to approximately 50% (middle bar). (B) The graph shows the result of a single experiment analysing the effect of different amounts of 16.4.1-GFP expression on Rev-CFP activity. Rev-CFP activity in the absence of 16.4.1-GFP was set at 100%. Coexpression of different amounts of 16.4.1-GFP reduced Rev activity to 35.1% (0.5 μg pC16.4.1sg143) or 12.8% (1 μg pC16.4.1sg143) demonstrating a dose dependent effect of the 16.4.1-GFP on Rev activity.

To investigate the influence of 16.4.1 on Rev function, we analysed the effect of IgG1-16.4.1 and 16.4.1-GFP fusion proteins on transactivation capacity of Rev using a previously described Rev-reporter assay [3]. The mRNA synthesized from the reporter gene in this assay contains a region coding for red fluorescent protein (RFP) and a non-coding region with HIV-1 derived sequence elements mediating Rev-responsiveness. These consist of multiple INS from the HIV-1 *gag *gene and the RRE from the HIV-1 *env *gene. Rev activity is measured by quantification of RFP reporter positive cells by flow cytometry using the gating strategy depicted in Fig. 8A (for further details see figure legend). Experiments were performed in 293T cells because of the high transfection efficiencies achieved in these cells.

The transactivation capacity of Rev in the absence of exogenous 16.4.1 was set at 100%. The result of 5 independent experiments demonstrate an approximately 50% reduction of Rev activity by coexpression of 16.4.1 fusion proteins (Fig. 8A). A dose-dependent effect of 16.4.1-GFP expression on Rev activity was observed (Fig. 8B). No effect was observed for numerous other gene products of a human cDNA-library tested in this assay (Wolff et al, manuscript in preparation).

**Figure 9:**
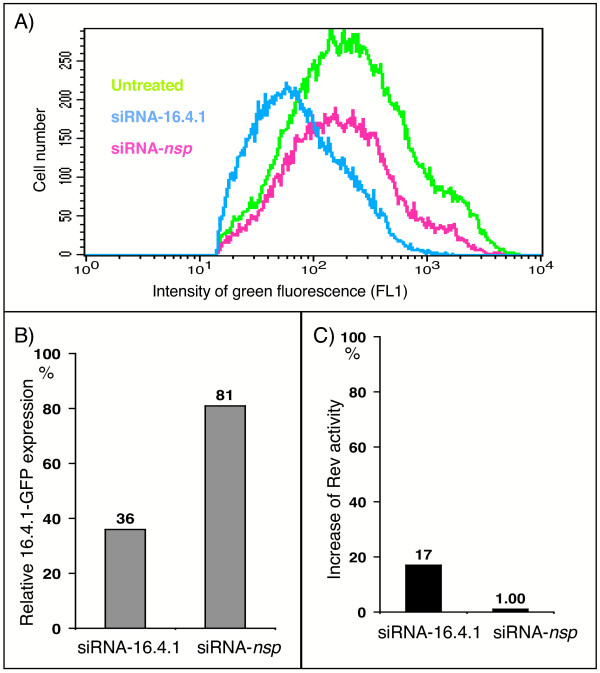
**Influences of endogenous 16.4.1 proteins on transactivation capacity of Rev in human cells**. (A and B) Downregulation of 16.4.1-GFP expression in HeLa cells by siRNAs. HeLa cells stably expressing 16.4.1-GFP were transfected with a pre-synthesized siRNA directed against a 16.4.1 specific sequence (siRNA-16.4.1) or a non-specific siRNA (siRNA-*nsp*). Expression of 16.4.1-GFP was quantified by flow cytometry in 40.000 cells. Panel A shows an overlay of GFP-expressing cell populations in untransfected cells (green curve) and after transfection with the 16.4.1-specific siRNA (blue) and the non-specific (red). 16.4.1-GFP expression in untransfected cells was set at 100% and the percentage of down regulation of green fluorescence indicated. siRNA-16.4.1 reduced expression levels of GFP-tagged 16.4.1 to 36% which was similar to the silencing effect of si-GFP which was analyzed as control (not shown). Higher levels of 16.4.1-GFP expression (81%) were observed in cells transfected with the unspecific siRNA-*nsp*. (C) Enhancement of Rev transactivation capacity by siRNAs targeted against 16.4.1. 293T cells were transfected with siRNA-16.4.1 or siRNA-*nsp *and Rev activities compared with mock-transfected cells (i.e. cells treated with the RNAi transfection reagent lacking RNA). Transfection of siRNA-16.4.1 increased Rev activity by 17% whereas the unspecific siRNA-*nsp *increased Rev activity by only 1%.

In the experiment above we showed that overexpression of 16.4.1-GFP exhibited a negative effect on the transactivation capacity of HIV-1 Rev in human cells. Isolation of 16.4.1 from a human cDNA library suggests that 16.4.1 proteins may be produced in human cells. To target expression of native 16.4.1 we decided to use RNA interference. To identify inhibitors of 16.4.1 expression we analysed several candidate siRNAs targeted to sequences within the 16.4.1 coding region and a negative control siRNA that recognizes sequences located upstream of the 16.4.1 coding region. An exemplary experiment is shown in Fig. 9A, B. HeLa 16.4.1-GFP cells were transfected with siRNAs and the effects on expression of 16.4.1-GFP monitored by flow cytometry (Fig. 9A and 9B). 16.4.1-GFP expression levels in RNAi transfected cells were determined relative to those in untransfected cells in 40.000 cells by FACS analysis. siRNA-16.4.1 reduced mean relative expression levels of 16.4.1-GFP to 36%. A similar effect was observed for a positive control siRNA that silences GFP (data not shown). The negative control siRNA (siRNA-*nsp*) only moderately diminished mean relative expression of 16.4.1-GFP to 81%. A similarly moderate reduction was observed for mock-transfected cells (data not shown) indicating that this is caused by the RNA transfection procedure. Analysis of the inhibitory effect of siRNA-16.4.1 on 16.4.1-GFP expression in three additional experiments yielded a mean relative expression of 16.4.1-GFP of 30.7% + 4.7 (standard deviation), confirming the inhibitory effect of this siRNA on 16.4.1-GFP.

Subsequently we investigated the effect of siRNA-16.4.1 and the negative control siRNA-*nsp *in 293T cells in the Rev-reporter assay described above (Fig. 9C). The negative control siRNA (siRNA-*nsp*) had no effect on Rev transactivation capacity compared to mock transfected cells (1% increase of Rev activity). In contrast, siRNA-16.4.1 increased Rev transactivation capacity by 17% compared to mock-transfected controls. A specific enhancing effect of siRNA-16.4.1 was observed in three independent experiments. These results indicate that endogenous 16.4.1 gene products are capable of modulating Rev activity.

### Expression of 16.4.1 proteins

Database searches identified several cDNAs of various lengths that contain the complete 16.4.1 sequence within a predicted open reading frame [For overview see additional file 1: Figure A1 and additional file 2: Table A1]. These are derived from various human tissues and cells. The predicted molecular masses of the hypothetical proteins encoded by these cDNAs range from ~145 kDa to 18.5 kDa. This suggests existence of several human 16.4.1 protein species encoded by various cDNAs, rather than a single 16.4.1 protein generated from a single cDNA.

Production of 16.4.1 proteins has not been reported so far and is currently under investigation in our laboratory (Kramer-Hämmerle et al., in preparation). In the context of this ongoing study, a monoclonal antibody (Mab) was generated against recombinant 16.4.1. Indirect immunofluorescence of HeLa cells transfected with the IgG1-16.4.1 expression plasmid revealed a cytoplasmic staining pattern that was indistinguishable from that obtained with antibodies against IgG1 [see additional file 3: Figure A2, A) a, for image]. The 16.4.1 Mab, but not the secondary antibody, also stained untransfected HeLa cells, yielding a cytoplasmic, granular pattern [see additional file 3, Figure A2, A) b for image]. In Western Blot analysis of HeLa-16.4.1-GFP cells, the 16.4.1 Mab recognized a band with the predicted molecular mass of ~45 kDa for the 16.4.1-GFP fusion protein as well as additional proteins [see additional file 3, Figure A2, B]. These results confirm specific recognition of 16.4.1 antigens by the 16.4.1 Mab and indicate expression of endogenous 16.4.1 proteins.

The 16.4.1 Mab has also been used to analyze cells from different lines and primary tissues, including brain and peripheral blood mononuclear cells. A staining pattern similar to that in HeLa cells was observed for 4 out 5 cell lines analyzed by indirect immunofluorescence (not shown). Western blot pagesanalysis of the cell lines/tissues investigated so far yielded a total of 4 distinct bands, ranging in size from > 150 kDa to < 30 kDa [for summary see additional file 3, Table A2]. The occurrence of these bands depended on the cell line/tissue investigated. These results confirm expression of 16.4.1 proteins in human cells and tissues and suggest cell-specific expression patterns of 16.4.1 proteins.

Future studies are directed at identifying the full range of 16.4.1 protein species with a panel of antibodies and characterizing 16.4.1 expression patterns on cDNA and protein levels in various cell types.

## Discussion

In this study we identified a cDNA encoding a novel cellular gene product (16.4.1) that interacts with the HIV-1 Rev protein in yeast and mammalian cells. In human cells, 16.4.1 is a substrate for CRM1-dependent export and shows predominant cytoplasmic localization. Colocalization of Rev and 16.4.1 was observed in nuclei, particularly in the nucleoli, of cells expressing both proteins. Overexpression and RNAi experiments indicate that 16.4.1 can influence transactivation function of Rev.

### Comparison of cytoplasmic localization properties of 16.4.1, Rev and PKI

We demonstrate that 16.4.1 interacts with CRM1 (Fig. 2) and shows similar Leptomycin B sensitive, cytoplasmic localization behaviour as PKIα and the carboxyterminal half of Rev (Fig. 3B) which are known substrates for CRM1-dependent export [53]. These results indicate that cytoplasmic localization of 16.4.1 involves CRM1-mediated nuclear export.

A nuclear export signal was mapped in 16.4.1 (Fig 5). Mutation of the Leucine/Isoleucine residues of the 16.4.1 NES only partially inhibited nuclear export of full-length 16.4.1, whereas leucine/isoleucine residues in the NES have been shown to be essential for export of Rev and PKIα [35,53]. This suggests differences in the export functions of the 16.4.1 NES and the NES of Rev and PKIα. This conclusion is supported further by the bioinformatics analysis, which showed that the group of NES sequences recognized by the same matrix as the 16.4.1 transport signal did not include the NES of Rev or PKIα (Fig. 6).

GFP fusion proteins containing a single copy of the 16.4.1 NES showed weaker cytoplasmic localization than GFP fusion proteins with tandem copies of this region or full-length 16.4.1-GFP (Fig. 4). This suggests that cytoplasmic localization of 16.4.1 does not depend solely on the functionality of a single copy of the 16.4.1 NES. The formation of homo-oligomers of 16.4.1, as shown by mammalian two-hybrid analysis (Fig. 2), could allow cooperative activity of multiple 16.4.1 NES. In addition, sequences beyond the NES could also contribute to cytoplasmic localization, for example by increasing cytoplasmic retention of 16.4.1. Sequences beyond the NES of 16.4.1 could also promote interactions with export-enhancing co-factors, several of which have been identified so far. These include the Ran-binding protein 3 (RanBP3) [54,55], NXT1 [56] and eukaryotic initiation factor 5A (eIF-5A). eIF-5A was demonstrated to be involved in export of Rev-like NES but not of the PKIα-NES [30,57], suggesting the existence of substrate-specific export cofactors. Future studies will be directed at identifying cellular interaction partners of the 16.4.1 protein and investigating their influence on its export activity.

### Interactions of 16.4.1 and Rev

In this study we show that 16.4.1 and Rev are capable of influencing biological properties of one another.

In cells expressing 16.4.1 and Rev, Rev can alter localization properties of 16.4.1 by recruiting 16.4.1 to the nucleus, in particular nucleoli. This is shown by colocalization of Rev and 16.4.1 in the nucleoli of cells expressing both proteins (Fig. 7). Cytoplasmic localization of 16.4.1 suggests that 16.4.1 interacts with Rev in the cytoplasm and is then translocated together with Rev to the nucleus and to nucleoli. The region of 16.4.1 that mediates interaction with Rev (amino acids 39–133, Fig. 1) contains the 16.4.1 NES (amino acids 86–105). Thus CRM1 could "bridge" interaction of 16.4.1 with Rev. CRM1-mediated interaction with Rev has been observed for several cellular proteins proposed to function as cofactors for nuclear export of Rev (see Background). However, amino acid positions of Rev essential for interaction with 16.4.1 are located outside the Rev NES, and an export-deficient NES-mutant of Rev (RevM10Bl) was capable of interacting with 16.4.1 (Fig. 1). This suggests that 16.4.1 does not function as an essential cofactor for nuclear export of Rev.

We have demonstrated that overexpression of 16.4.1 inhibits transactivation function of Rev (Fig. 8). The molecular mechanism underlying this inhibitory effect is unclear. A possible model to explain an inhibitory effect of 16.4.1 on Rev activity is that 16.4.1 recruited to nucleoli by Rev promotes association of Rev and CRM1 in inactive complexes. The strong interaction of 16.4.1 with CRM1 may increase the amount of CRM1 associated with Rev to inhibitory levels. In support of this model, experimental evidence has been obtained demonstrating that (1) Rev associates with CRM1 in nucleoli, influencing its mobility, (2) high levels of CRM1 inhibit Rev activity and (3) Rev is capable of recruiting other CRM1-interacting factors to nucleoli that are capable of inhibiting Rev activity [26,58,59]. This model will be investigated in future experiments.

The RNAi experiments suggest that endogenously expressed 16.4.1 gene products can also affect Rev function. As expected, the stimulatory effect of RNAi-mediated inhibition of 16.4.1 expression was small, since Rev is known to function efficiently in 293T cells [60]. We attempted to study the long-term effect of inhibition of endogenous 16.4.1 on Rev function by establishing cell lines stably expressing siRNA against 16.4.1. However, this approach was not feasible because of cell death after 2–3 weeks of expression of 16.4.1 siRNAs. This indicates that 16.4.1 gene products are crucial for cell viability. On the other hand, overexpression of 16.4.1 is well tolerated as demonstrated by the establishment of a cell line stably expressing 16.4.1-GFP.

The physiological role of interaction of Rev with 16.4.1 is not clear yet and may be positive or negative, depending on the levels of expression of 16.4.1. At low levels 16.4.1 proteins may act as a molecular chaperones of Rev, counteracting the strong tendency of Rev to aggregate with itself and/or preventing incorrect interactions with other cellular proteins. The occurrence of cytoplasmic cellular factors that inhibit Rev multimerization is suggested by a recent report demonstrating only weak Rev-Rev interaction in the cytoplasm of living cells [61]. At high concentrations, 16.4.1 may decrease transactivation function of Rev, for example by sequestering Rev in inactive complexes in nucleoli. Inactivation of Rev by 16.4.1 could play a role in protecting the cells from Rev-mediated cytotoxicity [62].

## Conclusion

HIV-1 infection of human cells involves various interactions between cellular and viral factors (reviewed in [63]). Some cell types (e.g. astrocytes) can control HIV-1 replication demonstrating the impact of cellular factors on HIV infection [60,64]. Identification of cellular factors that are able to interfere with viral replication will contribute to understanding of cellular defence mechanisms against viral intruders and may also lead to identification of new targets for therapeutic approaches for virus restriction. Using HIV-1 Rev as "bait" we were able to identify a previously undescribed cellular interaction partner of an HIV-1 protein, 16.4.1. The 16.4.1 protein is exported from the nucleus by CRM1 and accumulates in the cytoplasm. An important feature of 16.4.1 is its ability to impair transactivation capacity of Rev, although both proteins localize to different cellular compartments. Conversely, Rev is capable of affecting localization of the 16.4.1 protein by recruiting it to the nuclei/nucleoli of eukaryotic cells. Because of its properties we suggest naming the 16.4.1 protein "Risp" (Rev interacting shuttling protein).

Data base analyses and preliminary studies with a specific monoclonal antibody suggest that human proteins with Risp sequences are expressed in various human cell types including HIV-1 target cells. The objective of future studies will be to characterize Risp proteins, their cellular functions and their influence on Rev activity and HIV-1 replication in different HIV-1 target cells.

This study represents a further step toward elucidating the network of host cell factors that interact with the HIV-1 Rev protein and influence its functions. This study also illustrates the power of viral proteins as tools for identification and biological characterization of novel cellular factors. Use of similar experimental strategies as presented here will help to gain deeper understanding of virus-cell interactions.

## Methods

### Plasmids

The inserts of all plasmid constructs used in this study were verified by sequence analysis (Sequiserve, Germany).

#### Expression plasmids for yeast two-hybrid analysis

pEG202 [65] was used to express bait proteins containing various Rev sequences fused to the LexA DNA binding domain in yeast. pEG202 also expresses the yeast selection marker His3.

For construction of pEG202-sRev, pBsRev [64,66] was used as template for PCR amplification to generate the *rev *sequence of HIV-1 isolate HXB2; the PCR product was inserted into the EcoRI site of pEG202. The same procedure was used for construction of bait plasmids pEG202-RevM4, pEG202-RevM10BL, pEG202-RevM5, pEG202-RevSLT40, using pcTat-RevM4 [46], pCsRevM10BLsg143 [60], pcRevM5 [45] and pcRevSLT40 [46] as PCR templates. Bait plasmids used as controls for unspecific interaction contained the wildtype *rev *sequence in antisense orientation (pEG202-sRev(antisense)) or encode a bait protein unrelated to Rev (pEG202-LexCD2).

pJG4-5 and its derivative pJG4-6 was used for galactose-inducible expression of prey proteins containing the protein of interest fused to the NLS of SV40 T antigen, and the B42 transcriptional activator [65]. pJG4-5/6 also express the yeast selection marker Trp1.

pSH18-34 reporter plasmid contains 8 LexA operators that direct expression of the *lacZ *reporter gene.

The expression library used for two-hybrid screening contained cDNAs from human Jurkat T-cells inserted into the EcoRI/XhoI sites of pJG4-5 [67].

For mapping of Rev-interacting regions of 16.4.1, sequences encoding full-length 16.4.1 or various fragments of 16.4.1 were generated by PCR amplification using pC16.4.1sg143 as template and primers adding a 5' MluI site and a 3' NotI site. PCR products were inserted into pJG4-6 cleaved with MluI and NotI.

Plasmids pJG4-5, pJG4-6, pEG202, pSH18-34 and the Jurkat T-cell cDNA expression library were kindly provided by Waldemar Kolanus, University of Bonn, Germany.

#### Expression plasmids for mammalian two-hybrid analysis

Protein interactions in human cells were analysed with the CheckMate™ Mammalian Two-Hybrid System (Promega, Madison, USA), which uses pACT and pBIND vectors and the G5*luc *reporter plasmid. pACT and pBIND direct expression of fusion proteins containing the transcriptional activation domain of Herpes virus simplex VP-16 (pACT) or the Gal4 DNA binding domain (pBIND) at the N-terminus and potential interactor domains at the C-terminus. pG5*luc *contains 5 Gal4-binding motifs and a minimal promoter for inducible expression of the firefly luciferase reporter gene.

pACT and pBIND expression plasmids were constructed by PCR amplification of coding sequences from plasmid templates with primers adding restriction sites for insertion into the multiple cloning regions of the target vectors. The *rev *sequence was generated from pEG202-sRev and inserted into the SalI site of pACT (pACT-Rev). *16.4.1 *sequence was generated from clone DKFZp434O171Q (RZPD, Berlin, Germany;[68]) and inserted into the BamHI sites of pACT and pBIND (pACT-16.4.1 and pBIND-16.4.1). The human *CRM1 *sequence was amplified from pChCRM1sg143 [49] and inserted into the BamHI site of pACT (pACT-hCRM1).

#### Plasmids encoding GFP-tagged proteins

The vector pFRED143 contains a humanized version of a strong fluorescent GFP mutant (sg143) under the control of the CMV immediate early promoter [69]. pC-sg143 plasmids were constructed by using the cloning strategy described in [60], involving insertion of protein coding sequences without translational start and stop codons in frame with *gfp*-sequences into pFRED at a unique NheI site located immediately downstream of codon 1 of the GFP ORF.

The 16.4.1 sequence in pJG4-5 contains a 163 amino acid reading frame which is terminated by a stop codon but lacks an initiation codon. A potential translational initiation codon was identified 24 nucleotides upstream of and in frame with the 16.4.1 sequence in a human fetal cDNA (Gene Bank Accession Nr. W67699). For construction of pC16.4.1sg143, the 16.4.1 sequence in pJG4-5 was amplified with a 5' primer incorporating sequences encoding amino acids 2–7 into the PCR product, which was inserted into the NheI site of pFRED143. Sequence analysis of pC16.4.1sg143 verified formation of a single open reading frame by 16.4.1 and GFP sequences, resulting in expression of a protein with a predicted molecular mass of approximately 46 kDa.

For construction of pC16.4.1(74–133)sg143 the sequence encoding amino acids 74 to 133 was generated by PCR from pC16.4.1sg143 and inserted into the NheI site of pFRED143. pC16.4.1(74–133)_2_sg143 contains tandem sequences encoding amino acids 74 to 133 of 16.4.1.

The plasmid pC16.4.1(NESmut)sg143 expresses a mutant 16.4.1-GFP fusion protein in which L^92^, I^97 ^and I^99 ^within the core nuclear export of 16.4.1 are replaced by alanine residues. pC16.41(NESmut)sg143 was constructed by PCR based site directed mutagenesis from pC16.4.1sg143.

For construction of pCRev(52–116)sg143, sequences encoding amino acids 52–116 of Rev were amplified from pCsRevsg143 [60] and inserted into the BspEI site of the pFRED143 variant pFRED143BspEI.

pCPKIα sg143 directs expression of GFP-tagged human PKI and was constructed as described in [49].

#### Plasmids encoding IgG1-tagged proteins

pIg was kindly provided by Waldemar Kolanus and was used for construction of plasmids directing expression of 16.4.1 fusion proteins with an N-terminal IgG1 tag. pIg contains CH2 and CH3 domain segments from human IgG1 cDNA in the mammalian expression vector pRK5 [67,70]. Sequences encoding 16.4.1 or various regions of 16.4.1 were amplified from pC16.4.1sg143. PCR products were inserted into the MluI/NotI sites of the pIg polylinker, resulting in construction of the following plasmids: pIgG1-16.4.1, pIgG1-16.4.1(2–38), pIgG1-16.4.1(2–133), pIgG1-16.4.1(38–171), pIgG1-16.4.1(74–171) and pIgG1-16.4.1(134–171).

#### Plasmids used in Rev activity assay

pLRed(INS)_2_R reporter plasmid was constructed in a similar manner as described for pLRed(p17/p24INS)R [3]. Briefly, a DNA fragment with HIV-1 *gag*-sequences containing INS 1 and 2 (nucleotides 379–1424 of the HXB2 genome) was isolated from pB37R [71] by *Cla*I-digestion and inserted into *Cla*I-digested and dephosphorylated pLRedR [3]. pLRed(p17/p24INS)R contains two copies of the HIV-1 *gag *sequences in *sense *orientation. This plasmid directs Rev and Tat-dependent expression of red fluorescent protein (RFP).

pL3Tat contains the HIV-1 *tat *gene under the control of the HIV-1 LTR [64].

pCsRev-CFP was established by replacement of the GFP encoding sequence of pCsRevsg143 mentioned above with the coding sequence for cyan fluorescent protein (CFP).

### Yeast two-hybrid assay

The yeast interaction trap was performed essentially as described in [65,67], using yeast strain EGY48 which contains the *LEU2 *gene under the control of LexA operators and has defective *leu2*, *his3*, *trp1 *and *ura3 *genes. The pEG202-sRev bait plasmid was transformed into yeast strain EGY48 bearing the pSH18-34 reporter plasmid. This "bait" strain was transformed with the Jurkat T-cell library contained in the yeast expression plasmid pJG4-5. Criteria for protein-protein interactions were growth on medium containing galactose and lacking uracil, histidine, tryptophane and leucine, no growth in the same medium containing glucose instead of galactose, and expression of beta-galactosidase. Forty-six yeast transformants were obtained that grew on selective medium and expressed the *lacZ *reporter gene. cDNAs from those yeast clones were purified by passaging through E. coli KC8 and retested for interaction with pEG202-sRev but not with control bait plasmids pEG202-sRev(antisense) and pEG202-LexCD2 to confirm specific interaction.

### Mammalian two-hybrid assay

Mammalian two-hybrid assay was performed in HEK293 cells, using the CheckMate™ Mammalian Two-Hybrid System (Promega, Madison, USA).

HEK293 cells were cotransfected with pBIND and pACT constructs for expression of VP16 and Gal4 proteins fused to potential interactor domains (1 μg each) and with the pG5luc reporter plasmid (2 μg). For each interactor assay, parallel transfections were performed with G5luc and pBIND and pACT vectors expressing Vp16 and Gal4 without interactor domains to determine background expression of the luciferase gene. Two days after transfection cells were lysed, and firefly luciferase activity (in relative light units per second, RLU/s) quantified using the Luciferase Reporter Assay System (PROMEGA) and the ORION I Microplate Luminometer (Berthold Detection Systems, Pforzheim, Germany). The total amount of protein in cell lysates was quantified using the BCA Protein Assay Reagent Kit (Pierce Chemical Co., Rockford, USA) and luciferase activity (RLU/s) standardized to 1mg of total protein in the cell lysate. Values are expressed as fold-induction of luciferase activity over basal expression levels.

### Cell culture, transfection and Leptomycin B treatment

HeLa and HEK293 cells were maintained in Dulbecco's Modified Eagle Medium containing 2 nM Glutamax I (Life Technologies, Karlsruhe, Germany) and 10% fetal calf serum (Seromed, Berlin, Germany).

All transfection experiments were performed in 35-mm-diameter dishes (BD Biosciences, Bedford, MA, USA). Cells were seeded at a density of 1 × 10^5 ^cells per dish one day prior to transfection and cultured for 24 h after transfection. HEK293 cells were transfected by calcium phosphate coprecipitation using the CellPhect kit (Pharmacia, Freiburg, Germany). Transfection of HeLa cells was performed with the FuGENE™6 Transfection Reagent (Roche Diagnostics, Mannheim, Germany) using 500 ng plasmid DNA per dish.

Leptomycin B (LMB, Sigma-Aldrich, Munich, Germany) treatments were performed 24 hours after transfection at a concentration of 5 nM LMB for 2 hours. For microinjection experiments, LMB was added at a concentration of 10 nM 2 hours prior to injection.

For evaluation and quantification of fluorescence, cells were fixed with 4% paraformaldehyde (PFA) for 30 minutes at room temperature; nuclei were stained with Hoechst 33343 (Molecular Probes Europe BV, Leiden, Netherlands) for 10 minutes.

Toxic influences of long-term expression of 16.4.1-GFP in HeLa cells were assessed by CytoTox-ONE™ and CellTiter-Glo™ cell viability assays (Promega, Madison, USA) according to manufacturer's instructions.

The cell line HeLa 16.4.1-GFP expresses 16.4.1-GFP constitutively and was established by transfection with pC16.4.1sg143 followed by G418-selection (80 μg/ml). Non-fluorescent antibiotic resistant cells were excluded by FACS sorting.

### Immunological methods

#### Immunocytochemistry

Cells were fixed with PFA (4%) and permeabilized with TritonX-100 (0.2%).

IgG1-16.4.1 fusion proteins were detected by direct staining with a Cy3-conjugated goat anti-human IgG1 antibodies (Dianova, Hamburg, Germany), diluted 1:200 in phosphate buffered saline containing 1% BSA. For detection of 16.4.1 antigens, a monoclonal antibody against 16.4.1 (Kramer-Hämmerle et al. in preparation) was used as primary antibody and a Cy3-conjugated goat anti-rat antibody (Dianova, Hamburg, Germany) as secondary antibody.

#### Western Blot

Whole cell lysates were prepared with RIPA buffer containing protease inhibitors (protease inhibitor cocktail; Sigma, Taufkirchen, Germany) and separated on either precast 4–12% Bis-Tris or 3–8% Tris-Acetate gradient gels (Invitrogen, Karlsruhe, Germany). After transfer onto nitrocellulose membranes, proteins were probed with primary polyclonal rabbit antibodies against GFP (Chemicon Europe, Hofheim, Germany) or with the 16.4.1-specific monoclonal antibody and with HRP-conjugated secondary goat anti-rabbit or anti-rat antibodies (Dianova, Hamburg, Germany). Protein bands were detected by an enhanced chemiluminescence system (Perbio Science, Bonn, Germany).

### Quantitative fluorescence microscopy

Microscopy of cells expressing fluorescent proteins and quantitative analysis of subcellular distribution of fluorescence was performed as described [49]. Images for quantification were taken at 32-fold magnification with flexible exposure times and evaluated by IPlab software for fluorescence values below pixel saturation. Each cell group was photographed as phase contrast and fluorescence images for Hoechst 33342 and GFP.

### 3D deconvolution and widefield multichannel unmixing microscopy

Fluorescence microscopic imaging, 3D deconvolution and widefield multichannel unmixing was carried out with a computer-controlled Zeiss Axiovert 200 M research microscope ("Cell Observer") with scanning stage (Carl Zeiss, Goettingen, Germany) and Software AxioVision 4.2 (Carl Zeiss Vision, Hallbergmoos, Germany). Images of 2% PFA-fixed specimen were acquired using a Zeiss 40×/1.3 Plan Neofluar objective and Zeiss filter sets No. 44 (GFP), 49 (Hoechst33342) and 47 (CFP). Z-stacks with 100 optical sections at 0.325 μm intervals were captured with a Zeiss AxioCam HRm CCD-Camera with full resolution of 1388 × 1044 pixels. Deconvolution of fluorescence images [72] was carried out with AxioVision 4.2 software using a constrained iterative algorithm and auto linear normalization. Subsequently widefield multichannel unmixing was performed on the deconvolved image-stacks to correct for fluorescence bleedthrough of Hoechst-, CFP- and GFP- signals. Three reference samples with either one of the three fluorochromes were prepared, reference measurements were performed and a 3 × 3 matrix was generated that was used to unmix the sample image-stack. Processed images were then arranged for presentation and exported with AxioVision 4.2 software.

### Microinjection experiments

Compounds for microinjection were generated and microinjections were performed as described previously [49,51]. Briefly, bovine serum albumin (BSA) was first labeled with Alexa-red (excitation 568 nm, Molecular probes) and subsequently conjugated to the following peptides:

(1.) HIV-1 Rev-NES represented by -CGG-LQPPLERLTLD and (2.) 16.4.1 amino acids 86 to 105 -CG-KTLESNLFDDNIDIFADLTV. Both peptides were synthesized by Sigma-Genosys. For coinjections unconjugated BSA labeled with Alexa-green (excitation 488 nm, Molecular probes) was used. Peptide solutions with a concentration of 1 mg/ml were injected into the nuclear compartment of HeLa cells. Two hours after injection, cells were fixed with 4% PFA, images of green and red channels were taken and the percentages of red (BSA-conjugated peptides) and green (unconjugated BSA) fluorescent signals were determined. The ratio between red and green fluorescence in the nuclear and cytoplasmic compartments indicates the relative translocation activity of the peptide.

### Rev activity assay

The Rev activity assay was performed essentially as described previously [3]. Transfections were performed in 6-well plates using FUGENE™6 according to the manufacturer's protocol with 1 μg pLRed(INS)_2_R, 0.2 μg pL3Tat, 0.1 μg pCsRev-CFP and 0.1 μg pFRED143. To evaluate the influence of 16.4.1-GFP on Rev-activity, pC16.4.1sg143 (0.5 μg or 1 μg) was added to the transfection mixture. Expression of fluorescent fusion proteins was checked by microscopy 24 hours after transfection and cells were analysed by flow cytometry. Typically 100,000 cells of each transfected well were analysed with a Becton Dickinson FACSCalibur flow cytometer equipped with a 488 nm argon laser and controlled by the software CellQuest Pro. GFP-fluorescence was analysed in channel FL-1 and RFP-fluorescence in FL-3. The percentage of reporter positive cells (RFP) in the transfected cell population (GFP) was determined.

### RNAi interference in combination with Rev activity assay

For down-regulation of gene expression, 16.4.1 specific (siRNA-16.4.1) and non-specific (siRNA-*nsp*) and a GFP-specific siRNA were designed and synthesized by Qiagen. Transfections were carried out using RNAiFect transfection reagent (Qiagen) according to supplier's protocol. Target cells were seeded one day prior to transfection in 6-well plates, 5 μg siRNA per well were used for each transfection. Silencing effects of GFP-fusion proteins were determined by FACS analyses 48 hours after transfection. For the combined RNAi-Rev activity experiments, siRNA transfection was performed 24 h prior to DNA transfection.

### Bioinformatics

*In silico *identification and analysis of sequences were performed using the databases and bioinformatics tools of NCBI [73] and of Genomatix Software GmbH [74].

Similarities between nuclear export signals in the NES database NESbase 1.0 [75] were analysed with a set of amino acid weight matrices adapted from the MatInspector algorithm [76] using the BLOSSOM similarity matrix values to account for conserved amino acid substitutions. Reading frames were predicted with the tool ATGpr [77].

### Statistics

Statistical analysis of data was carried out with the GraphPadPRISM program (GraphPad Software Inc., San Diego, CA, USA). Significances of differences between data sets were determined by calculating two-tailed P values by the Mann-Whitney U test.

## Authors' contributions

SKH performed expression and localization studies of Risp; functional analyses of nuclear export of Risp and influences on Rev-activity and was primarily responsible for writing of the manuscript. FCS and CB carried out the yeast and mammalian two-hybrid analyses including design and construction of expression plasmids and final evaluation of the data. HW developed the Rev reporter assay, carried out 3D deconvolution and multichannel unmixing microscopy and participated in analysis of experimental data. MV performed the experiments regarding RNA interference. TW carried out the *in silico *analysis of the NES signals. VE contributed with substantial discussions and participated on interpretation of the results. RBW was the principal investigator and was responsible for designing the study, supervising the experimental work and for writing the manuscript together with SKH.

## Supplementary Material

Additional File 1 Schematic representation of cDNAs with 16.4.1-coding sequences in predicted open reading frames. The scheme shows various cDNAs with 16.4.1 sequences identified by BLAST search of Entrez databases ([73] (indicated by lines). Open reading frames (ORF) were predicted with the ATGpr program [77]. Bars indicate the locations of the predicted ORFs within the cDNAs. The positions of the potential translation initiation and stop codons are marked by M and *, respectively. Regions with 16.4.1 encoding sequences are labeled yellow. All accession numbers are from the GenBank database. Click here for file

Additional File 2 Sizes of hypothetical 16.4.1 cDNAs and proteins. The table indicates the lengths of the cDNAs shown in Additional file 1: Figure A1 and the calculated sizes of the proteins they are predicted to encode. Click here for file

Additional File 3 Analysis of expression of 16.4.1 proteins with a monoclonal antibody directed against 16.4.1. Figure A2 shows expression of 16.4.1 proteins in HeLa cells. (A) Immunohistochemical analysis. Expression of 16.4.1 was detected in HeLa cells transfected with pIgG-16.4.1 (panel a) and in non-transfected HeLa cells (panel b) by indirect immunofluorescence with the monoclonal antibody against 1.6.4.1 and Cy3-labelled secondary antibodies. Panel c shows lack of reactivity of non-transfected HeLa cells with the secondary antibody. All images were taken with the same exposure times (300 ms). (B) Western blot analyses. Additional to 16.4.1-GFP several proteins are detected in HeLa cells expressing 16.4.1-GFP after transfection of pC16.4.1sg143. Proteins in whole-cell lysates were separated by electrophoresis in gradient gels (4–12% and 3–8%), and blotted onto nitrocellulose membranes. 16.4.1 proteins were detected with the monoclonal 16.4.1 antibody and a secondary antibody conjugated with horse radish peroxidase (HRP). For detection of the 16.4.1-GFP fusion protein, the membranes were stripped and reprobed with polyclonal antibodies against GFP. Table A2 lists proteins recognized by the monoclonal antibody against 16.4.1 in different human cell lines and primary human tissues by Western blot analyses. HeLa (cervix carcinoma) and 293T (embryonal kidney) cell lines are non-neural cells and U138MG, U251MG and 85HG66 human glioblastoma cell lines. Human cortical brain tissue and peripheral blood mononuclear cells were also investigated.
Click here for file
