# Supplementary figures and images for: Identification of a novel Rev-interacting cellular protein
Source: BMC Cell Biol. 2005 Apr 24;6:20. doi: 10.1186/1471-2121-6-20 (PMC1097722; doi:10.1186/1471-2121-6-20)

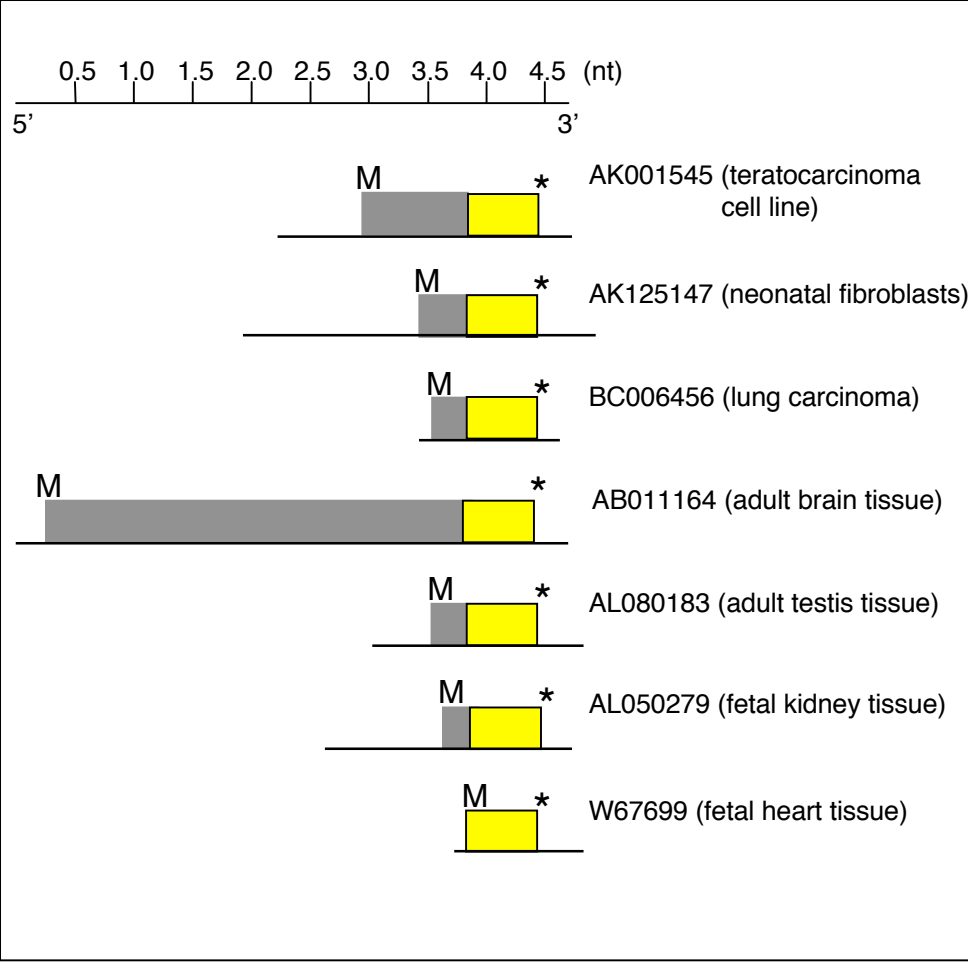

Supplement: Additional File 1 — Schematic representation of cDNAs with 16.4.1-coding sequences in predicted open reading frames. The scheme shows various cDNAs with 16.4.1 sequences identified by BLAST search of Entrez databases ([73] (indicated by lines). Open reading frames (ORF) were predicted with the ATGpr program [77]. Bars indicate the locations of the predicted ORFs within the cDNAs. The positions of the potential translation initiation and stop codons are marked by M and *, respectively. Regions with 16.4.1 encoding sequences are labeled yellow. All accession numbers are from the GenBank database. [file 1471-2121-6-20-S1.pdf]
