# Supplementary material for: Identification of a novel Rev-interacting cellular protein
Source: BMC Cell Biol. 2005 Apr 24;6:20. doi: 10.1186/1471-2121-6-20 (PMC1097722; doi:10.1186/1471-2121-6-20)
Supplement: Additional File 2 — Sizes of hypothetical 16.4.1 cDNAs and proteins. The table indicates the lengths of the cDNAs shown in Additional file 1: Figure A1 and the calculated sizes of the proteins they are predicted to encode. [file 1471-2121-6-20-S2.pdf]

Table A1. Analysis of cDNAs containing 16.4.1 coding sequences identified in Entrez database and their hypothetical proteins

Acc.#	cDNA (nt)	ORF (AA)	Predicted MW (kDa)
AK001545	2506	531	57
AK125147	3055	320	35
BC006456	1228	308	33
ABO11164	4623	1320	145
AL080183	1687	308	33
AL050279	2037	283	30.5
W67699	1101	164	18.5
