# Supplementary material for: Identification of a novel Rev-interacting cellular protein
Source: BMC Cell Biol. 2005 Apr 24;6:20. doi: 10.1186/1471-2121-6-20 (PMC1097722; doi:10.1186/1471-2121-6-20)
Supplement: Additional File 3 — Analysis of expression of 16.4.1 proteins with a monoclonal antibody directed against 16.4.1. Figure A2 shows expression of 16.4.1 proteins in HeLa cells. (A) Immunohistochemical analysis. Expression of 16.4.1 was detected in HeLa cells transfected with pIgG-16.4.1 (panel a) and in non-transfected HeLa cells (panel b) by indirect immunofluorescence with the monoclonal antibody against 1.6.4.1 and Cy3-labelled secondary antibodies. Panel c shows lack of reactivity of non-transfected HeLa cells with the secondary antibody. All images were taken with the same exposure times (300 ms). (B) Western blot analyses. Additional to 16.4.1-GFP several proteins are detected in HeLa cells expressing 16.4.1-GFP after transfection of pC16.4.1sg143. Proteins in whole-cell lysates were separated by electrophoresis in gradient gels (4–12% and 3–8%), and blotted onto nitrocellulose membranes. 16.4.1 proteins were detected with the monoclonal 16.4.1 antibody and a secondary antibody conjugated with horse radish peroxidase (HRP). For detection of the 16.4.1-GFP fusion protein, the membranes were stripped and reprobed with polyclonal antibodies against GFP. Table A2 lists proteins recognized by the monoclonal antibody against 16.4.1 in different human cell lines and primary human tissues by Western blot analyses. HeLa (cervix carcinoma) and 293T (embryonal kidney) cell lines are non-neural cells and U138MG, U251MG and 85HG66 human glioblastoma cell lines. Human cortical brain tissue and peripheral blood mononuclear cells were also investigated. [file 1471-2121-6-20-S3.pdf]

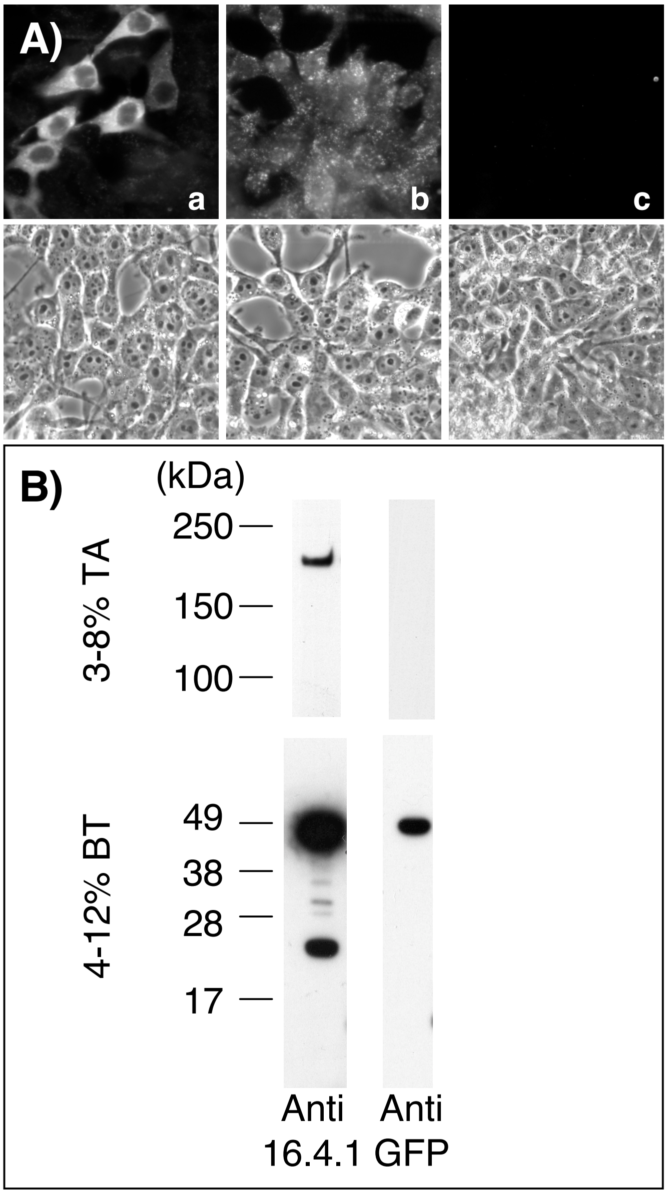

Table A2. Proteins detected with a 16.4.1-specific monoclonal antibody in different human cell lines and tissues

	>150 kDa	app. 45 kDa	app. 55 kDa	<30 kDa
HeLa, 293T	+	+	-	+
U138MG, U251MG, 85HG66	+	+	-	-
Human brain tissue, PBMC	-	-	+	-
